# Two new species of *Lactifluus* (Fungi, Russulales) from tropical *Quercus* forest in eastern Mexico

**DOI:** 10.3897/mycokeys.59.38359

**Published:** 2019-10-16

**Authors:** Leticia Montoya, Abraham Caro, Antero Ramos, Victor M. Bandala1

**Affiliations:** 1 Red Biodiversidad y Sistemática, Instituto de Ecología A.C., P.O. Box 63, Xalapa, Veracruz, 91000, México

**Keywords:** Ectomycorrhizal fungi, milkcaps new taxa, Neotropical fungi, oak forests

## Abstract

Two new species of Lactifluus subgenus Lactifluus were discovered during a three-year monitoring of the ectomycorrhizal fungi in a tropical oak forest from central Veracruz, Mexico. Systematic sampling of basidiomes allowed recording of the morphological variation of fruit-bodies in different growth stages along with their fructification season. Both new species were distinguished, based on macro- and micromorphological features and on molecular data. A phylogenetic analysis of a concatenated nuc rDNA ITS, D1 and D2 domains of nuc 28S rDNA (LSU) and the 6–7 region of the second largest subunit of the RNA polymerase II (*rpb*2) sequence dataset of species of *Lactifluus* is provided. In the phylogeny inferred, one of the new species is sister to *L. dissitus* Van de Putte, K. Das & Verbeken and the other belongs to the group of species of *L. piperatus* (L.) Kuntze, sister to an unidentified species from U.S.A. The studied taxa grow under *Quercus oleoides* in the study site. The species are presented and illustrated here.

## Introduction

Mexico is one of the worldwide centres for oak (*Quercus*) diversity. It hosts around 174 species, over 60% of which are endemic (Valencia 2004, Oldfield and Eastwood 2007, Villaseñor 2016). Most members of the genus grow in subtropical and temperate montane forests (1000–3500 m a.s.l.) and very few in lowland tropical areas (below 1000 m a.s.l.) (Valencia 2004). Some lowland tropical areas from central Veracruz (eastern Mexico) harbour oak forest patches, part of them being considered Pleistocene relicts and formally recognised amongst “main land regions” of the country (“Región Terrestre Prioritaria 104”) (Arriaga et al. 2000). These forests are important wildlife refuges, including fungi and endemic species of cycads and orchids (Castillo-Campos et al. 2005). The high biodiversity of the tropical oak forest and the ecosystem services provided are important for protecting prevailing relicts. Ectomycorrhizal (ECM) fungi, such as *Lactifluus* species, are undoubtedly a key to the growth and survival of *Quercus* seedlings and trees in such patches of native tropical forest under drought conditions and adverse edge effects, through greater water and nutrient absorption in degraded soils. Despite their importance, ECM fungi in such a Mexican ecosystem have received scarce taxonomic attention, excepting reports, from the area or surroundings, of a few Boletales, *Lactarius* s.l., *Cantharellus* (Guzmán and Sampieri 1984; García et al. 1987; Singer et al. 1991; Montoya and Bandala 1996, Herrera et al. 2018a, b).

The genus *Lactifluus* contains around 190 species based on Index Fungorum (http://www.indexfungorum.org) and recent publications and is widespread in a variety of ecosystems worldwide but with a clear predominance in the tropics, especially in tropical Africa, Asia and the Neotropical region (De Crop et al. 2017). Within subgenus Lactifluus, De Crop et al. (2017) recognised six sections molecularly and morphologically well-supported. Recent advances on the study of this genus in the tropics are indeed revealing high species diversity. For example, in at least two surveys related with *L. volemus* sensu lato, Van de Putte et al. (2010, 2012) discovered 24 phylogenetic species in a small area of northern Thailand and in Sikkim Himalaya. Dealing with section Piperati, the revision by De Crop et al. (2014), based on morphology and molecular data, threw light on its wide worldwide diversity and the possible existence of cryptic species. Moreover, they found that the European *L. glaucescens* and *L. piperatus* are not conspecific with species of the section from other regions.

In Mexico, around 19 species of *Lactifluus* (as *Lactarius*) have been recorded, most of them from montane (above 1200 m elevation) subtropical and temperate forests, in comparison with the higher proportion of surveys focused on the subtropical and temperate diversity in this country. In western Mexico, at elevations between 2200– 2550 m, the ECM community of *Quercus* spp. (including *Q. laurina* and *Q. crassifolia*), studied by Morris et al. (2008, 2009), included five species of milkcaps belonging to the genus *Lactarius* but none to *Lactifluus*. In our weekly monitoring of two tropical *Quercus* forests in eastern Mexico, we have noticed the presence of milkcaps, including *Lactifluus* species. One of our interests is to continue documenting their taxonomic identity and, in parallel with research, such as Herrera et al. (2018b), to provide morphological and molecular evidence of their association, at root tips level, with the native *Quercus* species. In this paper, we describe two new species found in these forests, recognised with morphological information and a multilocus phylogeny.

## Materials and methods

### Study area, sampling, morphological and colour study of basidiomes

Random visits were conducted during June-October of 2015–2017 to a remnant of the tropical *Quercus* forest from Central Veracruz (eastern Mexico). The site is privately owned, at Alto Lucero Co. (450–500 m elevation). Sampling of the two *Lactifluus* species studied was developed in monodominant stands of *Q. oleoides*, surrounded by a coffee trees plantation or land used for livestock.

Macro-morphological features and colours were recorded from fresh samples in different growth stages. Alpha-numeric colour codes in descriptions follow Kornerup and Wanscher (1967) (e.g. 7C8) and Munsell (1994) (e.g. 10YR 8/6). Basidiomes were dried with a hot air dehydrator (45 °C) over a week. Measurements and colours of micro-morphological structures were recorded in 3% potassium hydroxide (KOH) and Melzer´s solution. Methods to determine basidiospore ranges are those used by Montoya et al. (2019). Thirty five basidiospores per collection were measured (length and width of the spore in lateral view, excluding the ornamentation). These measurements are presented in taxonomic descriptions accompanied by the symbols: *X̄* representing the range of *X* (where *X* is the average of basidiospores length and width in each collection) and *Q̄* refers to the range of *Q* (where *Q* is the average of the ratio of basidiospore length/basidiospore width in each collection). The methods used to produce scanning electron microscope (FEI, Quanta 250 FEG.) images of their basidiospores are those used by Montoya and Bandala (2003). Twenty five basidia and cystidia per collection were measured. Line drawings were made with the aid of a drawing tube. Collections are part of the herbarium of the Institute of Ecology, A.C., Xalapa, Mexico (XAL) (Thiers B. [continuously updated] Index Herbariorum: a global directory of public herbaria and associate staff. New York Botanical Garden’s Virtual Herbarium. http://sweetgum.nybg.org/science/ih/ accessed June 2019).

### DNA extraction, PCR amplification and sequencing

Genomic DNA was extracted from fresh and dried basidiome tissue, according to Cesar et al. (2018). PCR was performed to amplify the nuc rDNA ITS (Internal Transcribed Spacer) and D1–D2 domains of nuc 28S rDNA (28S), using primers ITS1F and ITS5/ITS4 and LR0R/LR21 and LR7, respectively (Vilgalys and Hester 1990, White et al. 1990, Gardes and Bruns 1993). Regions 6 and 7 of the nuclear gene that encode the second largest subunit of RNA polymerase II (*rpb*2) were amplified with primers bRPB2 6f/fRPB2 7CR (Liu et al. 1999, Matheny 2005). The thermal cycler conditions for ITS and *rpb*2 markers were (i) initial denaturation at 95 °C for 5 min; (ii) 35 cycles of 30 sec at 95 °C, 30 sec at 55 °C and 40 sec at 72 °C (for LSU this condition was for 60 sec); and (iii) a 5 min final elongation at 72 °C. Amplified PCR products were sequenced using a Genetic Analyzer 3730XL (Applied Biosystems). Once sequences were assembled and edited, they were deposited at GenBank (Benson et al. 2017) and the accession numbers are indicated in Table 1.

**Table 1.** Fungal names, specimen vouchers, locations and GenBank accession numbers (for ITS, 28S and *rpb*2), with newly sequenced collections of *Lactifluus* subgenera *Lactifluus* and *Piperati* in bold.

Taxa	Voucher	Locality	ITS	LSU	*rpb2*
**Lactifluus sect. Lactifluus**					
*Lactifluus acicularis*	K. Van de Putte 08-029 – Type	Thailand	HQ318239	HQ318147	HQ328884
*Lactifluus corrugis*	AV05-290	USA	JN388976	JN388997	JN375600
*Lactifluus crocatus*	KVP08-035 – Type	Thailand	JN388985	HQ318152	HQ328889
*Lactifluus dissitus*	AV-KD-KVP09-134	India	JN388978	JN389026	JN375628
*Lactifluus distantifolius*	D. Stubbe 07-461 – Type	Thailand	HQ318223	HQ318124	HQ328866
*Lactifluus leptomerus*	AV-KD-KVP09-131 – Neotype	India	NR_119981	NG_060275	JN375625
*Lactifluus longipilus*	H.T. Le 168 – Type	Thailand	HQ318235	HQ318143	HQ328880
***Lactifluus mexicanus***	Montoya 5189	Mexico	MK211179	MK211188	MK258869
	Montoya 5266	Mexico	MK211180	MK211189	MK258870
	Montoya 5276 – Type	Mexico	MK211181	MK211190	MK258871
*Lactifluus pallidilamellatus*	Montoya 4716	Mexico	JQ753824	JQ348268	-
*Lactifluus oedematopus*	KVP 12-001 – Type	Germany	KR364100	KR364232	KR364319
*Lactifluus pinguis*	H.T. Le 117 – Type	Thailand	HQ318211	HQ318111	HQ328858
*Lactifluus subvolemus*	Kobeke Van de Putte 08-49 – Type	Slovenia	JQ753928	JQ348380	JQ348242
*Lactifluus versiformis*	AV-KD-KVP09-006 – Type	India	NR_119980	JN389033	JN375633
*Lactifluus vitellinus*	K. Van de Putte 08-024 – Type	Thailand	HQ318236	HQ318144	HQ328881
*Lactifluus volemus*	KVP 11-002	Belgium	JQ753948	KR364175	KR364360
**Lactifluus sect. Tenuicystidiati**					
Lactifluus aff. tenuicystidiatus	KUN:F75810	China	KC154105	KC154131	KC154157
*Lactifluus subpruinosus*	KUN:F73639 – Type	China	NR_155312	NG_060288	KC154161
*Lactifluus tropicosinicus*	KUN:F59627 – Type	China	NR_155322	NG_060321	KP347670
**Lactifluus sect. Gerardii**					
*Lactifluus atrovelutinus*	D.Stubbe 06-003	Malaysia	GU258231	GU265588	GU258325
*Lactifluus bicolor*	DS06-247	Malaysia	JN388955	JN388987	JN375590
*Lactifluus gerardii*	A.Verbeken 05-375	USA	GU258254	GU265616	GU258353
*Lactifluus genevievae*	G.G./D.K. 17-02-05 Type	Australia	GU258294	GU265657	GU258397
Lactifluus aff. ochrogalactus	AV-KD-KVP09-120	India	JN388956	JN388990	JN375593
*Lactifluus parvigerardii*	KUN:F61367 – Type	China	JF975641	NG_060270	JF975643
*Lactifluus petersenii*	A.Verbeken 05-300	USA	GU258281	GU265642	GU258382
**Lactifluus sect. Ambicystidiati**					
*Lactifluus ambicystidiatus*	KUN:F57008 – Type	China	NR_155311	NG_060287	KC154148
**Lactifluus sect. Allardii**					
*Lactifluus allardii*	J. Nuytinck 2004-008	USA	KF220016	KF220125	KF220217
**Lactifluus sect. Piperati**					
Lactifluus aff. glaucescens	AV 05-374	North America	KF220049	KF220150	KF220236
Lactifluus aff. piperatus	A. Verbeken 04-202	USA	KF220021	KF220127	KF220220
	A. Verbeken 05-295	USA	KF220048	KF220149	KF220235
	A. Verbeken 05-393	USA	KF220050	KF220151	KF220237
	H.T. Le 198	Thailand	KF220099	KF220194	KF220268
	H.T. Le 242	Thailand	KF220100	KF220195	KF220269
	H.T. Le 293	Thailand	KF220101	KF220196	KF220270
	H.T. Le 378	Thailand	KF220102	KF220197	KF220271
	H.T. Le 51	Thailand	KF220076	KF220175	KF220253
	J. Nuytinck 2011-036	Vietnam	KF220105	KF220200	KF220274
	J. Nuytinck 2011-072	Vietnam	KF220106	KF220201	KF220275
	TENN 064342	USA	KR364103	KR364234	KR364324
*Lactifluus dwaliensis*	H.T. Le 67	Thailand	KF220108	KF220203	KF220277
*Lactifluus leucophaeus*	A.Verbeken 97-382 – Type	Papua New Guinea	GU258299	GU265640	GU258379
***Lactifluus lorenae***	Caro103	Mexico	MK211187	MK211196	MK258874
	Montoya 5190 – Type	Mexico	MK211185	MK211194	MK258872
	Montoya 5191	Mexico	MK211186	MK211195	MK258873
*Lactifluus piperatus*	A. Fraiture 2584	Belgium	KF220080	KF220176	KF220254
	J. Vesteholt 96-144	Denmark	KF220081	KF220177	KF220255
	M. Lecomte:2000 10 07 01	France	KF220033	KF220135	KF220225
	R. Walleyn 25-08-92b	Germany	KF220082	KF220178	KF220256
	UE09.08.2004-6	Sweden	DQ422035	DQ422035	DQ421937
	GENT:78111 – Type	France	KF220122	KF220215	-
*Lactifluus roseophyllus*	J. Nuytinck 2011-076	Vietnam	KF220107	KF220202	KF220276
**Outgroup**					
*Auriscalpium vulgare*	PBM 944	North America	DQ911613	DQ911614	AY218472
*Bondarzewia montana*	AFTOL 452	No data	DQ200923	DQ234539	AY218474
*Stereum hirsutum*	AFTOL 492	No data	AY854063	AF393078	AY218520

### Phylogenetic methods

Following preliminary analyses that placed the new species within Lactifluus subgenus Lactifluus, phylogenetic analyses were performed with the newly generated sequences and the sequences retrieved from GenBank (Benson et al. 2017) derived from the BLAST search (best match) of related *Lactifluus* species, complemented with other GenBank sequences of species of all the sections within Lactifluus subgenus Lactifluus, considered by De Crop et al. (2017) (Table 1). We constructed a concatenated sequence dataset (ITS+LSU+*rpb*2 sequences), with final length of 2,423 bp, in PhyDE v.0.9971 (Müller et al. 2010), aligned with MUSCLE algorithm (Edgar 2004) and corrected inconsistencies manually. Using the IQ-Tree (Nguyen et al. 2015) in an interface online (Trifinopoulos et al. 2016), we calculated the evolutionary model with a partitioning analysis (Kalyaanamoorthy et al. 2017; Chernomor et al. 2016) and Edge-unlinked partition model (Lopez et al. 2002), using the Bayesian Information Criterion (BIC), the Akaike Information Criterion (AIC) and corrected AIC to select the best-fit model. This later was used to generate a phylogenetic tree with the Maximum Likelihood (ML) method, with a Nearest Neighbour Interchange (NNI) heuristic, with TNe+I+G evolutionary model and Ascertainment Bias Correction (ASC). We also generated a consensus tree, calculating the Robinson-Foulds distance between the ML tree and the consensus tree, the branches being tested by means of Ultrafast Approach Bootstrap (UFBoot), SH-like approximate Likelihood Ratio Test (SH-aLRT), Approximate Bayes test (aBayes) and Bootstrap Standard (BS). A phylogenetic tree was generated also by Bayesian Inference (BI), using MrBayes v. 3.2.6 (Ronquist et al. 2012). The phylogenies from ML and BI analyses were displayed using FigTree v1.4.3 (Rambaut 2016).

## Results

**Figure 1.**
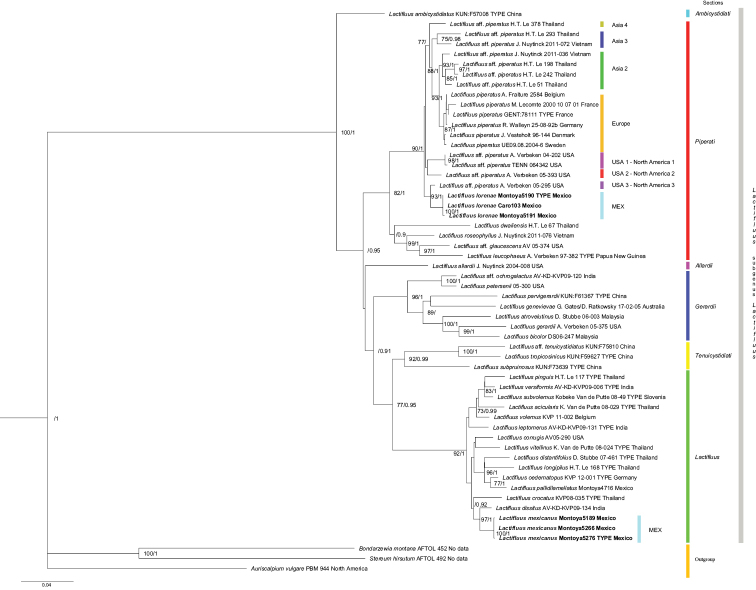
Concatenated three-locus (nuc rDNA ITS, nrLSU and *rpb*2) phylogenetic analysis by maximum likelihood of *Lactifluus* species. Bootstrap scores (only values ≥ 70) / Posterior probabilities (only values ≥ 0.90) are indicated above branches. New species are indicated in bold letters.

We generated 18 new sequences from *Lactifluus* species studied, six from each of ITS, nLSU regions of rDNA and *rpb*2 (Table 1 and alignment deposited in TreeBASE S23676). The dataset built included a total of 54 sequences and *Auriscalpium vulgare*, *Bondarzewia montana* and *Stereum hirsutum* as the outgroups. In the phylogenetic trees, inferred using both ML and BI, terminal clades were concordant amongst topologies and internal nodes that had significant BS score (≥ 70%), BI (≥ 0.90), UFBoot (≥ 95%), aBayes (≥ 0.90) and SH-aLRT (≥ 80%). The ML tree with the two former values for the nodes is presented here (Fig. 1). The generated sequences from the Mexican specimens clustered with strong support in two terminal clades.

Based on morphological features and supported with the grouping displayed in the phylogenetic tree, we recognised two groups of the Mexican samples studied representing two distinct new species of *Lactifluus*. One of them, *Lactifluus mexicanus*, appears sister (with strong support) to *L. dissitus* from India and the other one, *L. lorenae*, clusters in a clade with *L. piperatus* (L.) Kuntze from Europe and related species from North America and Asia, sister (with strong support) to an undescribed species from U.S.A.

### Taxonomy

Below, we present a key to facilitate the morphological recognition of the species here described. It is based on information from the specimens studied and on research dealing with subgenus Lactifluus (Hesler and Smith 1979; Verbeken and Horak 1999; Das et al. 2003; Van de Putte et al. 2010, 2012, 2016; De Crop et al. 2014, 2017).

Basidiomes staining brown or brownish when bruising or cut, especially the lamellae, context and latex; pleurolamprocystidia present **II. Sect. Lactifluus**

Basidiomes not staining as above; pleuromacrocystidia present **I. Sect. Piperati**

### I. Sect. Piperati

**Table:** 

1	Lamellae pink salmon to pale orange-brownish	***L. roseophyllus***
–	Lamellae whitish or cream colour	**2**
2	Pileus brownish grey; latex drying bluish-green	***L. leucophaeus***
–	Basidiomes whitish	**3**
3	Lamellae distant; latex white, slowly becoming light greenish-yellow on exposure	***L. dwaliensis***
–	Lamellae crowded	**4**
4	Basidiomes staining orange-brown when bruised; basidiospores with *Q̄* = 1.20–1.27; Pleuromacrocystidia 40–53 µm length	***L. lorenae***
–	Basidiomes not staining orange-brown; basidiospores more ellipsoid, with *Q̄* = 1.26–1.40; pleuromacrocystidia 50–90 length μm	**5**
5	Basidiospores with *Q̄* = 1.28–1.40, may form incomplete reticulum; suprapellis 80–120 μm thick; lamellae margin heterogeneous, cheilomacrocystidia 35–55 × 5–10 μm	***L. piperatus***
–	Basidiospores with *Q̄* = 1.26–1.33, ornamentation never forming a reticulum; suprapellis 10–30 μm; lamellae margin almost composed of emergent cheilomacrocystidia 55–70 × 7–9 μm	***L. glaucescens***

### II. Sect. Lactifluus

**Table:** 

1	Lamellae moderately distant to distant	**2**
–	Lamellae close or crowded	**4**
2	Smell mild	***L. oedematopus***
–	Smell of seafood	**3**
3	Interlamellae distance a relation of up to 5L+l/cm; basidiospores ornamentation up to 2.1 μm high; pleurolamprocystidia 45–155 × 5–7 µm; wall up to 3 µm thick; Cheilolamprocystidia 25–90 × 4–5.5 μm	***L. distantifolius***
–	Interlamellae distance denser (up to 8L+l/cm); basidiospores ornamentation up to 1.7 (−1.8) μm high; pleurolamprocystidia 60–145 × 7–9(−10) μm; wall up to 4 (−4.5) µm thick; Cheilolamprocystidia 15–80 × (4-) 6–10 μm	***L. dissitus***
4	Lamellae crowded (interlamellae distance a relation of up to 35L+l/cm)	***L. leptomerus***
–	Lamellae with a less dense arrangement	**5**
5	Odour mild	**6**
–	Odour of seafood	**7**
6	Pileus surface smooth to rugose; basidiospores (7.7–)7.8–9.9(−10.1) μm wide	***L. versiformis***
–	Pileus surface clearly wrinkled, even merulioid or with gyrose-reticulate wrinkles; basidiospores wider (8.5–)9–11(– 12) µm wide	***L. corrugis***
7	Pileus in pale and dull colours	**8**
–	Pileus more pigmented with darker or brighter tonalities, pileus including orange, brown, reddish or vinaceous colours	**9**
8	Pileus colour pale brownish-yellow	***L. subvolemus***
–	Pileus pale yellowish-white or straw-coloured	***L. pinguis***
9	Pileus mostly reddish-brown to vinaceous, brown with pinkish tinges	**10**
–	Pileus mostly in yellowish-orange to orange-brown tinges	**11**
10	Stipe with pinkish-orange, pinkish-brown tinges; suprapellis elements and pleurolamprocystidia up to 63 µm long; basidiospores ornamentation up to 1.5 μm high	***L. mexicanus***
–	Stipe brownish-orange; suprapellis elements up to 130 μm long, thus pileus surface with a more velvety appearance; pleurolamprocystidia up to 115 μm long; basidiospores ornamentation up to 2.3 μm high	***L. longipilus***
11	Basidiomes mostly in light yellowish-orange or orange tinges; basidiospores ornamentation up to 2(–2.4) high	**12**
–	Basidiomes with orange colouration but including darker brown colours; basidiospores ornamentation shorter	**13**
12	Basidiospores with a *Q̄* = 1.10–1.14; pleurolamprocystidia 64–120 × 6.4 – 9.6 µm; pileipellis terminal elements 16–40.8 × 2.4–12.8 μm	***L. pallidilamellatus***
–	Basidiospores with a *Q̄* = 1.07–1.09; pleurolamprocystidia 55–105 × 6–13 μm; pileipellis terminal elements 10–70 (–85) × 5–15 μm	***L. vitellinus***
13	Pileipellis terminal elements 10–70(–75) × 4–11 μm	***L. crocatus***
–	Pileipellis terminal elements slender up to 100–130 × 2.5–8 μm	**14**
14	Basidiospores 7.7–11.3 × 7.1–10.3 (–10.6) µm; pleurolamprocystidia 55–145(–160) × (6–)7–12 µm; cheilolamprocystidia 20–115 µm long	***L. volemus***
–	Basidiospores 7.0–9.1(–9.3) × 6.5–8.5 μm; pleurolamprocystidia 35–100 × 6–9(–11.5) μm; cheilolamprocystidia 15–85 μm long	***L. acicularis***

#### Lactifluus lorenae

Taxon classificationFungi

Montoya, Caro, Ramos & Bandala sp. nov.

4449E347-64B4-5FFA-986F-F68B7B80B064

829060

**Figure 2.**
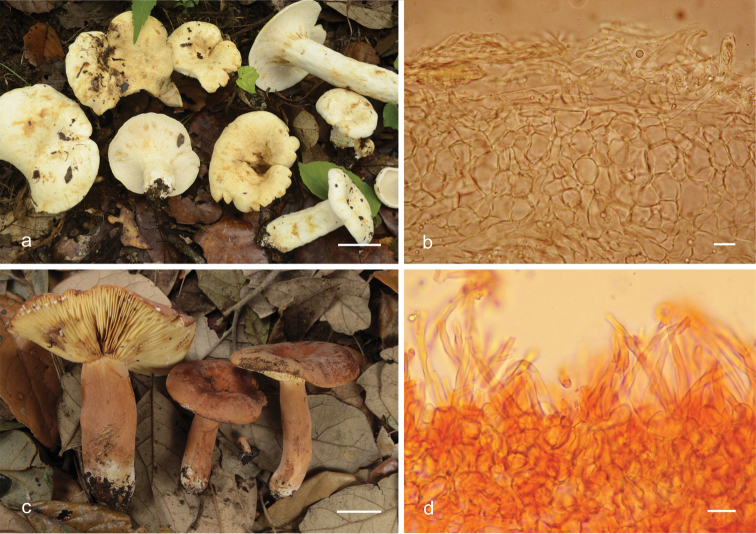
*Lactifluus* species basidiomes and pileipellis **a, b***L. lorenae*; **c, d***L. mexicanus*. Scale bars: 40 mm (**a**), 20 mm (**c**), 2 μm (**b, d**).

**Figure 3.**
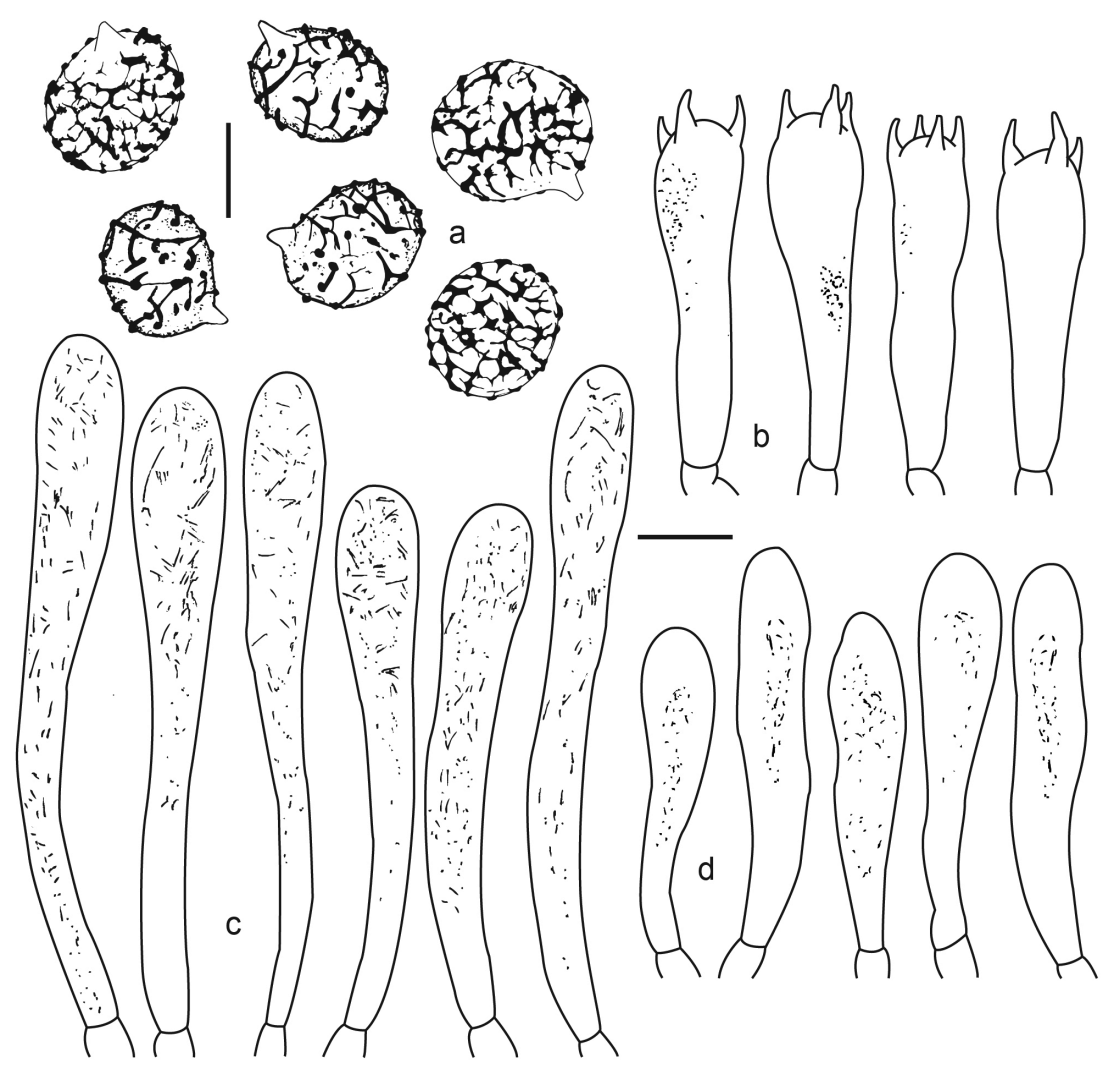
*Lactifluus lorenae* microscopical characteristics **a** basidiospores **b** basidia **c** pleurocystidia **d** cheilocystidia. Scale bars: 5 µm (**a**), 10 µm (**b–d**).

Figs 2a, b , 3 , 5a, b

##### Holotype.

MEXICO, Veracruz State, Alto Lucero Co., 12 km SW Palma Sola (road Veracruz-Nautla) 25 June 2015, Montoya 5190 (XAL). Ectomycorrhizal, under *Quercus oleoides*.

##### Diagnosis.

*Lactifluus lorenae* is clearly distinguished by white basidiomes, staining orange-brown, latex staining white paper yellow, odour somewhat chlorine-like, basidiospores broadly ellipsoid, pleuromacrocystidia 40–53 × 7–9 µm and pileipellis a hyphoepithelium with a gelatinzied hyphoid layer, 30–60 µm wide.

##### Gene sequences ex-holotype.

MK211185 (ITS), MK211194 (LSU), MK258872 (*rpb*2).

##### Etymology.

In honour of Dr. Lorena E. Sánchez Higueredo because of her interest in the conservation of tropical oak forest relicts in Veracruz, Mexico.

**Pileus** 25–114 mm diam., convex when young, expanded to broadly infundibuliform, undulate, depressed at centre when old, smooth to irregular when old, dull whitish with yellow tinges (3A2–3A5), staining orange-brown (5C6–C7) when bruised; margin decurved when young, with edge faintly decurved to straight when old, continuous to irregular. **Lamellae** adnate to subdecurrent, crowded to very close, 0.5–1.8 mm broad, edge entire, bifurcate at different levels, yellowish (3–4A2), staining orange-brown when handled, with lamelullae of different sizes, approximately 1 lamelullae per two lamellae. **Stipe** 20–90 × 11–35 mm, eccentric, cylindrical, attenuated or broadened towards the base, robust but at times flattened; surface smooth to irregular, faintly velvety under lens, more evident towards the base, whitish to cream-white, with yellow stains (5Y8/6), staining orange-brown when handled. **Context** cream colour, changing to brownish-orange when exposed, compact. Odour somewhat like chlorine; taste acrid. **Latex** whitish, milky, at times somewhat serous, staining white paper yellow (5Y 8/2), brownish after some minutes; taste burning acrid. KOH staining the pileus and stipe yellow to pale reddish.

**Basidiospores** (6–)6.5–8(–10) × (5–)5.5–6.5(–9) µm; *X̄* = 7.0–7.3(–9.2) × 5.5–6.0(–7.6) µm; *Q̄* = 1.20–1.27, broadly ellipsoid, thin-walled; ornamentation 0.2–0.4 µm high (measured under SEM), an incomplete reticulum, composed of thick and thin bands and some isolated warts, others ornamented almost with isolated warts and some unconnected bands, plage inamyloid; under SEM the relief of the bands of the basidiospores ornamentation appear with an irregular inflated shape and the plage area with reminiscences of ornamentation. **Basidia** 30–45 × 8–11 µm, clavate, some subcylindrical, with refractive contents, thin-walled, with 2, 4 or at times 3 sterigmata. **Pleuromacrocystidia** 40–53 × 7–9 µm, clavate, some cylindrical and faintly broadened towards the middle area, thin-walled, with refractive needle-like and granular contents. **Cheilomacrocystidia** 34–54 × 7–9 µm, cylindrical, some clavate at base, thin-walled, with refractive contents. **Pseudocystidia** absent. **Pileipellis** a hyphoepithelium; suprapellis layer of 30–60 µm thick, gelatinized, composed of periclinally orientated hyphae, in some areas the hyphae are loosely intermixed or at times projected in mounds of up to 85 µm thick, the gelatinized matrix dissolved in KOH after some minutes; hyphae 2–4 µm broad, cylindrical, septate, wall up to 0.5 µm thick, sinuous; subpellis of 50–130 µm thick, composed of subisodiametric cells, 12–35 × 10–38 µm diam., yellowish in KOH, wall up to 1.0 µm thick; dermatocystidia 37–128 × 6–8 µm, 3.6–4.8 µm diam. at base, clavate, with refractive needle-like and granular contents, wall up to 0.5 µm thick, scarce, arising from subisodiametric cells of the subpellis layer. **Context** hyphae 5–7 µm broad, cylindrical, thin-walled, some with walls 0.5 µm thick, with faint refractive contents, sphaerocytes 12–26 µm diam., pale yellowish, wall 0.5(–1) µm thick, frequent, laticiferous hyphae 4–7 µm diam., infrequent. **Hymenophoral trama** composed of hyphae which are 4–6 µm diam., septate, wall 0.5 µm thick, with sphaerocytes of 10–25 µm diam., pale yellowish, wall 0.5 µm thick, laticiferous hyphae 4–6 µm diam., infrequent. Clamp connections absent.

##### Habitat.

Gregarious, under *Quercus oleoides*, infrequent.

##### Additional studied material.

MEXICO, Veracruz, Alto Lucero Co., 12 km SW Palma Sola (road Veracruz-Nautla) 25 June 2015, Corona 1127, Montoya 5191; October 11, 2016, Caro 103 (all at XAL).

#### Lactifluus mexicanus

Taxon classificationFungi

Montoya, Caro, Bandala & Ramos sp. nov.

21BD75FD-A2F3-52BF-8897-7BE7B29C5270

829061

**Figure 4.**
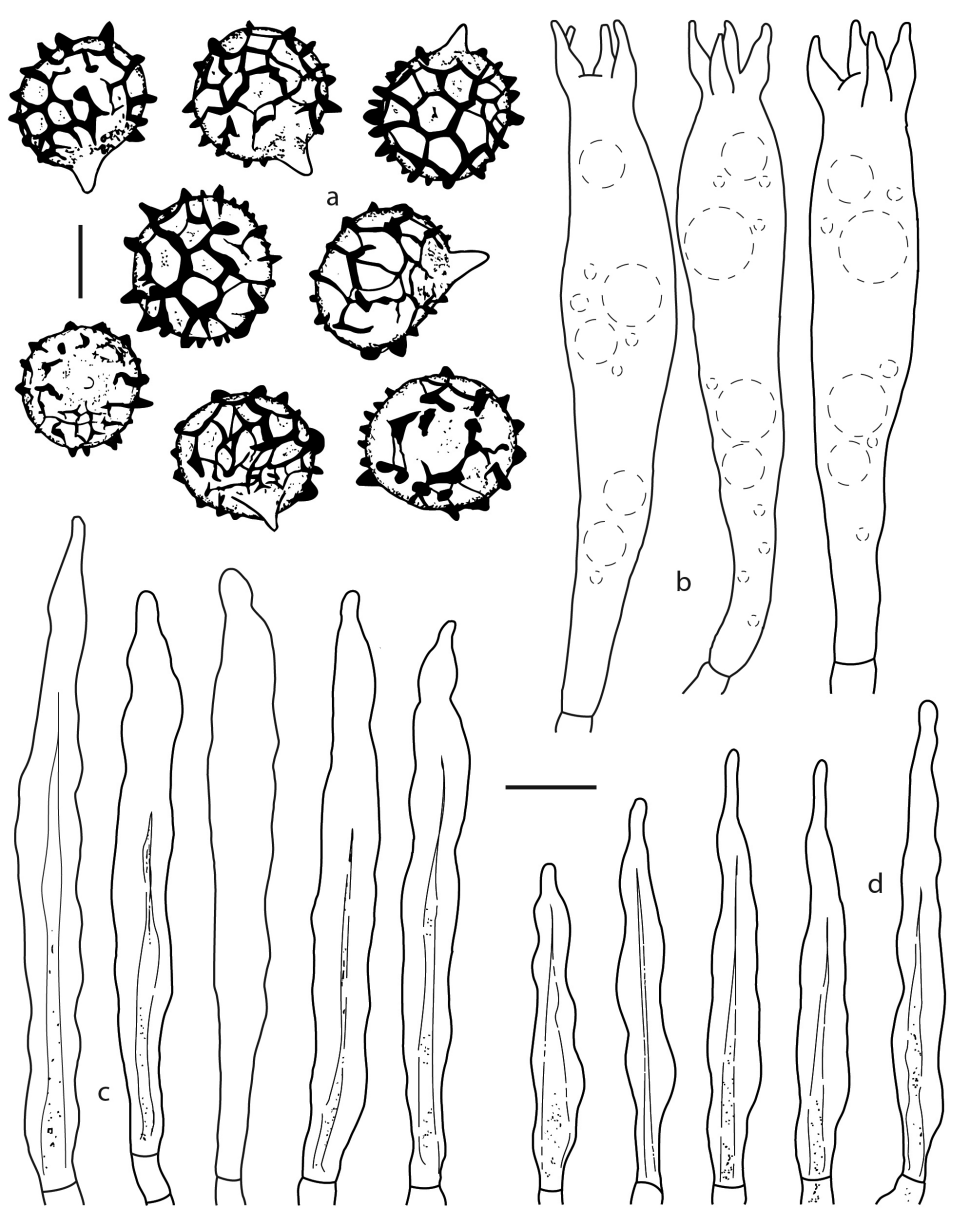
*Lactifluus mexicanus* microscopical characteristics **a** basidiospores **b** basidia **c** pleurocystidia **d** cheilocystidia. Scale bar: 5 µm (**a**), 10 µm (**b–d**).

**Figure 5.**
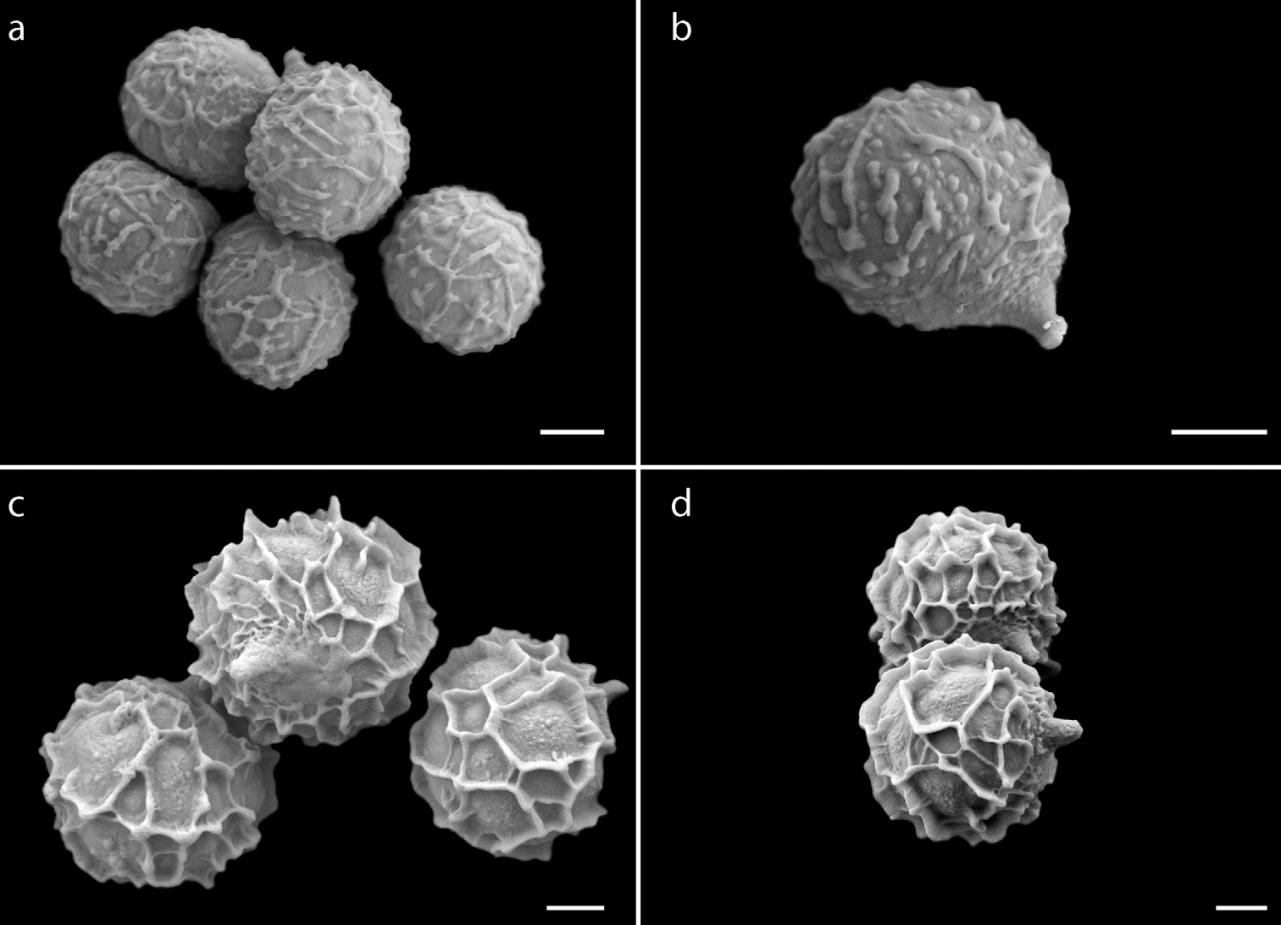
SEM microphotographs of *Lactifluus* species **a, b***Lactifluus lorenae***c, d***L. mexicanus*. Scale bar: 2 μm.

Figs 2c, d , 4 , 5c, d

##### Holotype.

MEXICO, Veracruz State, Veracruz, Alto Lucero Co., 12 km SW Palma Sola (road Veracruz-Nautla) 11 July 2016, Montoya 5276 (XAL). Ectomycorrhizal, under *Quercus oleoides*.

##### Diagnosis.

Recognised by the combination of pileus disc faintly rugose, margin rugose to strongly venous-rugose, lamellae close to very close, the stipe including pinkish tinges and by the size of lamprocystidia and pileipellis terminal elements.

##### Gene sequences ex-holotype.

MK211181 (ITS), MK211190 (LSU), MK258871 (*rpb*2).

##### Etymology.

referring to Mexico.

**Pileus** 33–125 mm diam., convex, plano convex to depressed at centre, subvelvety, smooth or at times faintly rugose at centre, at remaining disc surface smooth, vinaceous-brown or vinaceous (7D6–8; 7E8; 8C7; 8D4–8) when young, then ferruginous-brown, cinnamon-brown, frequently pale vinaceous (7C4–6), dull vinaceous (7D6) or pinkish-wine over a yellowish base, other reddish-brown to vinaceous (7C8–E8, 7D7–8; 2.5YR 4–5/6), at times with orange-brown (6C7; 6D7–8; 5YR 5/6–6/6; 7.5YR 5/4, 5/6–8) areas; margin decurved, straight in age, at times undulated, rugose to strongly venous-rugose. **Lamellae** 2–9 mm broad, close to very close, adnate to subdecurrent, arcuate, with entire edge, some furcate at different levels, at times sinuous especially towards the stipe attachment, pale yellowish to yellowish (2.5Y 8/1–3, 8/6; 7.5YR 8/4; 10YR 8/3–6), straw-yellow, yellow-orange (4A2–6 surfaces, 5A3–5 edges in group) with brown to cinnamon-brown tinges, with faint vinaceous stains or brown colour (2.5YR 5/3; 7.5YR 5/4) when handled; lamelullae of different sizes, 1–4 per lamellae. **Stipe** 35–115 × 9–27 mm, cylindrical, faintly broadened towards the base, subtomentose, dry, solid, in general concolorous but paler than pileus surface, at apex pale pinkish-orange (5YR 8/3–4), pinkish-brown, pale orange-brown or pinkish-red (6B3–4, 6B6–C6; 5YR 7/4–6, 8/2), continuing in pale orange (6A2–3), brown-orange with pinkish-grey tinges (6B2–5, 6C2) and pinkish-brown (6–7B3, 6–7B4) colours, becoming darker towards the base (7C4–6) (2.5YR 4/6; 5YR 6/4; 5YR 6/6; 7.5YR 6/3, 5/4, 8/4, 8/6), with some dark brown areas; base whitish and with whitish mycelium. **Context** compact, whitish to yellowish, staining brown-vinaceous. Odour faintly disagreeable, fishy; taste mild to somewhat bitter. **Latex** whitish to cream colour (2.5Y 8/3–6), milky, abundant, secreting from the whole basidiome, staining the lamellae and white paper pale brown; taste mild. KOH darkens the pileus surface.

**Basidiospores** 8–10(–11) × 7–9(–10) µm, *X̄*= 8.7–9.2 × 7.5–9.0 µm, *Q̄* = 1.1–1.2, subglobose to broadly ellipsoid, thin-walled; ornamentation up to 0.2–1.2 µm high (measured under SEM), a rather complete reticulum with irregular ridges, at times with thin connecting lines, rarely with some isolated ridges; plage in most spores inamyloid, rarely faintly amyloid; under SEM, the basidiospores wall appears rugose and with some isolated verrucae, with a complete reticulum composed of continuous regular or irregular ridges, some parts of the reticulum having rounded or irregular nodulose elevations, these later seen in the light microscope as verrucae, plage area smooth or with ornamentation reminiscences. **Basidia** 38–47 × 8–13 µm, clavate to faintly cylindrical, with 3–4 sterigma (at times with 2), thin-walled, with refractive contents. **Pleurolamprocystidia** 47–63 × 5–8 µm, lanceolate, at times mucronate, with wall 1.0–2.0 (–3.0) µm thick (in some elements, the wall is so thick that the lumen is very narrow). **Cheilolamprocystidia** 40–55 × 5–8 µm, lanceolate, some subcylindrical, at times mucronate, with wall up to 1.0 µm thick, without dense contents, hyaline. **Pseudocystidia** absent. **Pileipellis** a lampropalisade, elements of the suprapellis 45–63 × 3–6 µm, most cylindrical, others clavate, ventricose or even ovoid 10–12 × 5–6 µm, without dense contents, some septate, hyaline, compact, at times, the elements arranged in mounds, wall up to 0.5 µm thick; subpellis 42–70 µm thick, composed of cells 9–30 × 7–20 µm, inflated, some subisodiametric, others irregular in form, wall 0.5–1.0 µm thick, not gelatinzied, pale yellowish in KOH. **Context** hyphae in an irregular arrangement, 5.0–8.0 µm diam., cylindrical, septate, wall up to 0.5 µm thick, laticiferous hyphae 4–7 µm diam., with refractive contents, yellowish in KOH; sphaerocytes 14–20 × 16–22 µm, yellowish, wall 1–1.5 µm thick scarce. **Hymenophoral trama** with hyphae 4–8 µm diam., cylindrical, septate, wall up to 0.5 µm thick, with scarce refractive contents, intermixed with laticiferous hyphae 4–8 µm diam., with refractive contents, yellowish in KOH; sphaerocytes 19–27 µm diam., hyaline, with a faint yellowish tinge. Clamp connections absent.

##### Habitat.

Solitary or gregarious, under *Quercus oleoides*.

##### Additional studied material.

MEXICO, Veracruz, Alto Lucero Co., 12 km SW Palma Sola (road Veracruz-Nautla) 25 June 2015, Montoya 5189, 5192; 3 July 2015, Montoya 5193; 5 July 2016, Montoya 5266; 4 October 2016, Montoya 5294, 5295; 29 June 2017, Caro 109, Montoya 5329, 5330, 5331; 4 July 2017, Corona 1370, 1371; 10 July 2017 Montoya 5340; 12 September 2017, Montoya 5398; 16 September 2017, Montoya 5411, 5412; 19 September 2017, Caro125, 126; 25 September 2017 Corona 1423, 1424 (all at XAL).

## Discussion

The results inferred in the multilocus phylogeny (Fig. 1), strongly support the recognition of the two new species, *Lactifluus lorenae* and *L. mexicanus*. Although we faced difficulties to amplify *rpb*2 region, fortunately, the Mexican collections processed allowed us to recover with success, this and also ITS and 28S regions. The resolution obtained in our phylogeny may be related to the vouchers selection, mostly having sequences of the three regions (ITS, 28S and *rpb*2). The strong support of the clades, especially of *L. mexicanus* and *L. lorenae* allow us to complement morphological results and, on this basis, we decided to describe them. Both species are members of subgenus Lactifluus, the first one falling in section Piperati and the second in section Lactifluus, according to the classification proposed by De Crop et al. (2017).

*Lactifluus lorenae* is a white milkcap, with basidiomes showing macromorphological similarities with *L. piperatus*, as narrowly cicumscribed by De Crop et al. (2014). When comparing the macro- and micromorphological variation displayed in the Mexican samples and the information provided by De Crop et al. (2014) about *L. piperatus* in the strict sense, significant differences between the two taxa are detected. Basidiomes of the Mexican species show a uniform tendency to develop an orange-brown colouration on the surfaces when handled and in the context when exposed. The latex can be somewhat serous, staining white paper yellow and becoming brownish after some minutes. When comparing micromorphological features between *L. lorenae* and *L. piperatus* (according to the later authors), in the former, the basidiospores are more globose (*Q̄* = 1.20–1.27 vs. *Q̄* = 1.28–1.40) and pleurocystidia are distinctly shorter (40–53 × 7–9 µm vs. 50–70(–90) × 8–11 μm). Another difference between the taxa is the pileipellis structure, which in the Mexican species presents a thicker hyphoid suprapellis (30–60 µm thick vs. 10–30 μm thick) and with abundant dermatocystidia in the suprapellis in *L. piperatus*, while scarce in the subpellis in the Mexican taxon. Organoleptic differences may be noted between both taxa too, because in *L. lorenae*, the odour is somewhat like chlorine, while in *L. piperatus*, it is slightly acidic, distinctly honey- or apple-like when drying. In the inferred phylogeny (Fig. 1), *L. lorenae* clusters sister to an unidentified species, L. aff. piperatus USA 3-North America 3, but unfortunately, there is no information available on its morphological features and habitat from the U.S.A. to compare with the Mexican species.

*Lactifluus mexicanus* can be recognised by the combination of close to very close lamellae, pileus in vinaceous, reddish-brown, ferruginous-brown and pinkish-wine tinges, with a paler stipe, mostly including pinkish-orange to pinkish-brown tinges, short cystidia and pileipellis terminal elements (Pleurolamprocystidia 47–63 × 5–8 µm, cheilolamprocystidia 40–55 × 5–8 µm, terminal cells 45–63 × 3–6 µm). *Lactifluus mexicanus*, is recovered as sister species of *L. dissitus* from India, this latter differs by having more distant gills arrangement and clearly larger cystidia [pleurocystidia: 60–145 × 7.0–9 (−10) μm vs. 47–63 × 5–8 µm; cheilocystidia: 15–80 × (4–) 6–10 μm vs. 40–55 × 5–8 µm] (Van de Putte et al. 2012). *Lactifluus mexicanus* is a macro- morphological look-alike of the American *L. corrugis* (Peck) Kuntze. According to the original description of *L. corrugis* (Peck 1880), for which sequences of the type specimen are not available and based on information by Hesler and Smith (1979), the two species share the velvety cap surface and, to some extent, the general basidiome colour. However, in the latter, the basidiomes tend to be darker, especially the stipe (“… at times tinged reddish brown”) and the pileus surface is definitively more conspicuously wrinkled, even “...merulioid or corrugated with gyrose-reticulate wrinkles...”. In *L. mexicanus*, the cap surface at the disc centre is smooth or only faintly rugose, with the remaining surface smooth, except for the margin, which may appear rugose to strongly venose-rugose, but never with the merulioid aspect depicted in *L. corrugis*. Based on the information of Hesler and Smith (1979), micromorphological differences between both taxa also exist. *Lactifluus mexicanus* has shorter and narrower basidiospores [8–10 (–11) × 7.0–9.0 (–10) µm vs. 9–12 × (8.5–)9–11(–12) µm], with the basidiospore ornamentation up to 1.5 µm high vs. (0.2–) 0.4–0.7 (–0.8) µm high in *L. corrugis*. The cystidia and pileipellis terminal elements are shorter in the Mexican species (pleurocystidia: 4– 63 × 5–8 µm vs. (48–) 60–125 (–204) × 6–10 (16) µm; cheilocystidia: 40–55 × 5.0–8.0 µm vs. (25–) 35–78 × (2) 4–8 µm; pileipellis terminal elements 45–63 × 3–6 µm vs. 45–80 (–128) × 2.5–6 µm]. The pleurocystidia in *L. corrugis*, according to Hesler and Smith (1979), even have a thicker wall up to 7 µm thick. Moreover, this latter species appears to have a more temperate habit, growing in deciduous and mixed woods in U.S.A.

From the weekly sampling in tropical *Quercus* forest, during 2015–2017, we conclude that basidiomes of the studied species are produced in June-October, with those of *Lactifluus mexicanus* being more abundant. Although close to other edible species (Boa 2004, Borah et al. 2018), we have no records of edibility for *L. mexicanus* in the area.

Considering the high diversity of *Quercus* and *Pinus* species in Mexico, they represent important ECM hosts, related with the milkcaps in the country. *Quercus oleoides*, with a wide distribution from Mexico to Costa Rica, especially represents a key ECM host for this group of fungi in its range. In Costa Rica, however, at an elevation around 215 m, associated with *Q. oleoides*, Desai et al. (2016) found 37 ECM species belonging to different genera, three of which were determined as *Lactarius* but no *Lactifluus* was recorded. Considering that the two *Lactifluus* species, here studied, were found in a monodominant area of *Q. oleoides*, we consider them as putative mycobionts of this tree species. However, this will need to be confirmed at root tip level with molecular evidence, as in other milkcaps, such as *Lactarius trichodermoides* Montoya, Bandala & M. Herrera and *L. subplinthogalus* Coker (Herrera et al. 2018b). The two latter species associate with *Q. sapotifolia* and *Q. glaucescens*, respectively, in the relicts of the tropical oak forests from central Veracruz, Mexico.

## Supplementary Material

XML Treatment for Lactifluus lorenae

XML Treatment for Lactifluus mexicanus

## References

[B1] Arriaga L Espinoza JM Aguilar C Martínez E Gómez L Loa E (2000) Regiones terrestres prioritarias de México. Comisión Nacional para el Conocimiento y uso de la Biodiversidad. México.

[B2] Benson DA Cavanaugh M Clark K Karsch-Mizrachi I Lipman DJ Ostell J Sayers EW (2017) GenBank. Nucleic Acids Research 45 (Database issue): D37–D42. 10.1093/nar/gkw1070 PMC5210553 27899564

[B3] Boa E (2004) Wild edible fungi a global overview of their use and importance to people. FAO, Rome.

[B4] Borah N Semwal RL Garkoti SC (2018) Ethnomycological knowledge of three indigenous communities of Assam, India. Indian Journal of Traditional Knowledge 17: 327–335.

[B5] Castillo-Campos G Medina-Abreo ME Dávila PD Zavala JA (2005) Contribución al conocimiento del endemismo de la flora vascular en Veracruz, México. Acta Botánica Mexicana 73: 19–57. 10.21829/abm73.2005.1004

[B6] Cesar E Bandala VM Montoya L Ramos A (2018) A new *Gymnopus* species with rhizomorphs and its record as nesting material by birds (*Tyrannideae*) in the subtropical cloud forest from eastern Mexico. MycoKeys 42: 21–34. 10.3897/mycokeys.42.28894 PMC6262046 30510469

[B7] Chernomor O Haeseler A Minh BQ (2016) Terrace aware data structure for phylogenomic inference from supermatrices. Systematic Biology 65: 997–1008. 10.1093/sysbio/syw037 27121966 PMC5066062

[B8] Das K Sharma JR Verbeken A (2003) New species of *Lactarius* from Kumaon Himalaya, India. Mycotaxon 88: 333–342.

[B9] De Crop E Nuytinck J Van de Putte K Lecomte M Eberhardt U Verbeken A (2014) *Lactifluus piperatus* (Russulales, Basidiomycota) and allied species in Western Europe and a preliminary overview of the group worldwide. Mycological Progress 13: 493–511. 10.1007/s11557-013-0931-5

[B10] De Crop E Nuytinck J Van de Putte K Lecomte M Eberhardt U Verbeken A (2014) *Lactifluus piperatus* (Russulales, Basidiomycota) and allied species in Western Europe and a preliminary overview of the group worldwide. Mycol Progress 13: 493–511. 10.1007/s11557-013-0931-5

[B11] De Crop E Nuytinck J Van de Putte K Wisitrassameewong K Hackel J Stubbe D Hyde KD Halling R Moreau PA Eberhardt U Verbeken A (2017) A multi-gene phylogeny of *Lactifluus* (*Basidiomycota*, *Russulales*) translated into a new infrageneric classification of the genus. Persoonia 38: 58–80. 10.3767/003158517X693255 29151627 PMC5645188

[B12] Desai NS Wilson AW Powers JS Mueller GM Egerton-Warburton1 LM (2016) Ectomycorrhizal diversity and community structure in stands of *Quercus oleoides* in the seasonally dry tropical forests of Costa Rica. Environmental Research Letters 11: 125007. 10.1088/1748-9326/11/12/125007

[B13] Edgar R (2004) MUSCLE: Multiple Sequence Alignment with High Accuracy and High Throughput. Nucleic Acids Research 32: 1792–1797. 10.1093/nar/gkh340 15034147 PMC390337

[B14] García J Castillo J Guzmán G (1987) Segundo registro de *Boletellus jalapensis* en México. Biotica 12: 291–295.

[B15] Gardes M Bruns D (1993) ITS primers with enhanced specificity for basidiomycetes application to the identification of mycorrhizae and rusts. Molecular Ecology 2: 113–118. 10.1111/j.1365-294X.1993.tb00005.x 8180733

[B16] Guzmán G Sampieri A (1984) Nuevos datos sobre el hongo comestible *Cantharellus odoratus* en México. Boletín de la Sociedad Mexicana de Micología 19: 201–205.

[B17] Herrera M Bandala VM Montoya L (2018a) *Cantharellus violaceovinosus*, a new species from tropical *Quercus* forests in eastern Mexico. MycoKeys 32: 91–109. 10.3897/mycokeys.32.22838 PMC5904560 29681739

[B18] Herrera M Bandala VM Montoya L (2018b) Two Lactarius species (subgenus Plinthogalus) in ectomycorrhizal association with tropical *Quercus* trees in eastern Mexico. Mycologia 110: 1033–1046. 10.1080/00275514.2018.1521685 30481132

[B19] Hesler LR Smith AH (1979) North American Species of *Lactarius*. University of Michigan, Ann Arbor.

[B20] Kalyaanamoorthy S Minh BQ Wong TKF Haeseler A Jermiin LS (2017) ModelFinder: Fast model selection for accurate phylogenetic estimates. Nature Methods 14: 587–589. 10.1038/nmeth.4285 28481363 PMC5453245

[B21] Kornerup A Wanscher JH (1967) Methuen Handbook of Colour. 2^nd^ edn. Methuen, London, 243 pp.

[B22] Liu YJ Whelen S Hall BD (1999) Phylogenetic relationships among ascomycetes: evidence from an RNA polymerase II subunit. Molecular Biology and Evolution 16: 1799–1808. 10.1093/oxfordjournals.molbev.a026092 10605121

[B23] Lopez P Casane D Philippe H (2002) Heterotachy, an Important Process of Protein Evolution. Molecular Biology and Evolution 19: 1–7. 10.1093/oxfordjournals.molbev.a003973 11752184

[B24] Matheny PB (2005) Improving phylogenetic inference of mushrooms with RPB1 and RPB2 nucleotide sequences (*Inocybe*; Agaricales). Molecular Phylogenetics and Evolution 35: 1–20. 10.1016/j.ympev.2004.11.014 15737578

[B25] Montoya L Bandala VM (1996) Additional new records on *Lactarius* from Mexico. Mycotaxon 57: 425–450.

[B26] Montoya L Bandala VM (2003) Studies on *Lactarius* a new combination and two new species from Mexico. Mycotaxon 85: 393–407.

[B27] Montoya L Garay-Serrano E Bandala VM (2019) Two new species of *Phylloporus* (Fungi, Boletales) from tropical *Quercus* forests in eastern Mexico. MycoKeys 51: 107–123. 10.3897/mycokeys.51.33529 31139006 PMC6520330

[B28] Morris MH Pérez-Pérez MA Smith ME Bledsoe CS (2008) Multiple species of ectomycorrhizal fungi are frequently detected on individual oak root tips in a tropical cloud forest. Mycorrhiza 18: 375–383. 10.1007/s00572-008-0186-1 18704515

[B29] Morris MH Pérez-Pérez MA Smith ME Bledsoe CS (2009) Influence of host species on ectomycorrhizal communities associated with two co-occurring oaks (*Quercus* spp.) in a tropical cloud forest. FEMS Microbiology Ecology 69: 274–287. 10.1111/j.1574-6941.2009.00704.x 19508503

[B30] Müller J Müller K Neinhuis C Quandt D (2010) PhyDE – Phylogenetic Data Editor, version 0.9971. Program distributed by the authors. http:\\www.phyde.de

[B31] Munsell Soil Colour Charts (1994) Macbeth, New Windsor, 10 pp.

[B32] Nguyen LT Schmidt HA Haeseler A Minh BQ (2015) IQ-TREE: A Fast and Effective Stochastic Algorithm for Estimating Maximum-Likelihood Phylogenies. Molecular Biology and Evolution 32: 268–274. 10.1093/molbev/msu300 25371430 PMC4271533

[B33] Oldfield S Eastwood A (2007) The Red List of Oaks. Flora and fauna International, Cambridge, UK.

[B34] Peck Ch (1880) Annual Report New York State Museum Natural History 32: 31 pp. [1879]

[B35] Rambaut A (2016) FigTree v1.4.3 software. Institute of Evolutionary Biology, University of Edinburgh. http://tree.bio.ed.ac.uk/software/figtree/

[B36] Ronquist F Teslenko M van der Mark P Ayres DL Darling A Höhna S Larget B Liu L Suchard MA Huelsenbeck JP (2012) MrBayes 3.2: efficient Bayesian phylogenetic inference and model choice across a large model space. Systematic Biology 61: 539–542. 10.1093/sysbio/sys029 22357727 PMC3329765

[B37] Singer R García J Gómez LD (1991) The Boletineae of Mexico and Central America III. J. Cramer, Stuttgart. Nova Hedwigia, Beihefte, 102 pp.

[B38] Trifinopoulos J Nguyen LT Haeseler A Minh BQ (2016) W-IQ-TREE: a fast online phylogenetic tool for maximum likelihood analysis. Nucleic Acids Research 44(W1): W232–W235. 10.1093/nar/gkw256 PMC4987875 27084950

[B39] Valencia S (2004) Diversidad del género *Quercus* (Fagaceae) en México. Boletín de la Sociedad Botánica de México 75: 33–53. 10.17129/botsci.1692

[B40] Van de Putte K Nuytinck J Stubbe D Le HT Verbeken A (2010) *Lactarius volemus* sensu lato (Russulales) from northern Thailand: morphological and phylogenetic species concepts explored. Fungal Diversity 45: 99–130. 10.1007/s13225-010-0070-0

[B41] Van de Putte K Nuytinck J Das K Verbeken A (2012) Exposing hidden diversity by concordant genealogies and morphology – a study of the *Lactifluus volemus* (Russulales) species complex in Sikkim Himalaya (India). Fungal Diversity 55: 171–194. 10.1007/s13225-012-0162-0

[B42] Van de Putte K Nuytinck J De Crop E Verbeken A (2016) *Lactifluus volemus* in Europe: Three species in one Revealed by a multilocus genealogical approach, Bayesian species delimitation and morphology. Fungal Biology 120: 1–25. 10.1016/j.funbio.2015.08.015 26693681

[B43] Verbeken A Horak E (1999) *Lactarius* (Basidiomycota) in Papua New Guinea. 1. Species of Tropical Lowland Habitats. Australian Systematic Botany 12: 767–779. 10.1071/SB98026

[B44] Vilgalys R Hester M (1990) Rapid genetic identification and mapping of enzymatically amplified ribosomal DNA from several Cryptococcus species. Journal of Bacteriology 172: 4239–4246. 10.1128/jb.172.8.4238-4246.1990 PMC213247 2376561

[B45] Villaseñor JL (2016) Checklist of the native vascular plants of Mexico. Revista Mexicana de Biodiversidad 87: 559–902. 10.1016/j.rmb.2016.06.017

[B46] White TJ Bruns TD Lee SB Taylor JW (1990) Amplification and direct sequencing of fungal ribosomal RNA genes for phylogenetics. In: Innis MA Gelfand DH Sninsky JJ White TJ (Eds) PCR protocols: a guide to methods and applications.Academic Press, San Diego, 315–322. 10.1016/B978-0-12-372180-8.50042-1

